# Development and validation of HPLC-MS^2^ methodology for the accurate determination of C4–C8 B-type flavanols and procyanidins

**DOI:** 10.1038/s41598-021-93993-0

**Published:** 2021-07-20

**Authors:** Ugo Bussy, Yusuf Olanrewaju, Alan Crozier, Javier Ottaviani, Catherine Kwik-Uribe

**Affiliations:** 1 Mars Incorporated, McLean, VA USA; 2 Department of Nutrition, University of California Davis, Davis, CA USA; 3 Department of Chemistry, King Saud University, Riyadh, Saudi Arabia

**Keywords:** Mass spectrometry, Dietary carbohydrates, Small molecules

## Abstract

Cocoa flavanols and procyanidins (CFs), natural dietary bioactives, have been studied extensively over the past two decades for their potential health benefits. Research on their safety and efficacy is critically dependent upon on the ability to reliably characeterize the research materials that are utilized, and with growing consumer availability of CF-based products, reliable methods for the detection of potential adulteration are of increasing importance. This research focused on the development of a high performance liquid chromatography-tandem mass spectrometry method (HPLC-MS^2^) using primary standards and ^13^C-labelled procyanidins as internal standards. The ability of MS^2^ detection to discriminate A- and B-type procyanidins was demonstrated. Method performances were validated for degrees of polymerization up to four in seven model food matrices. Accuracy ranged from 90.9 to 125.4% and precision was < 10% at lower concentrations. Finally, the method was applied to cocoa-based samples and compared to the AOAC 2020.05 analytical protocol, supporting the use of NIST 8403 as reference material for HPLC-MS^2^ analysis.

## Introduction

There is a growing scientific and consumer interest for plant-derived bioactives in the human diet as their intake is thought to mediate positive health effects. Examples of dietary bioactives include (poly)phenols such as flavanols and their polymeric derivatives, the procyanidins. Flavanols and procyanidins occur in many foods, typically together, making their chemical analysis challenging. In the context of the diet, foods such as apples, cocoa^1,2^, nuts, grapes and berries^3^ can be particularly rich in flavanols and procyanidins Specifically, cocoa flavanols (CFs) represent a group of flavanols and procyanidins that have been subjected to extensive research in recent years which has revealed beneficial effects on cardiovascular health and cognitive performance^4–7^. Further developing accurate and reliable methods for the quantification of flavanols and procyanidins becomes essential in order to properly characterize materials used in human research and to better assess their levels and distribution in the diet^8,9^. Furthermore, with growing consumer interest in CF-based products, the ability to reliably determine the authenticity of products, including the potential adulteration with other flavanol and procyanidin sources, is of increasing importance.

**Figure 1:**
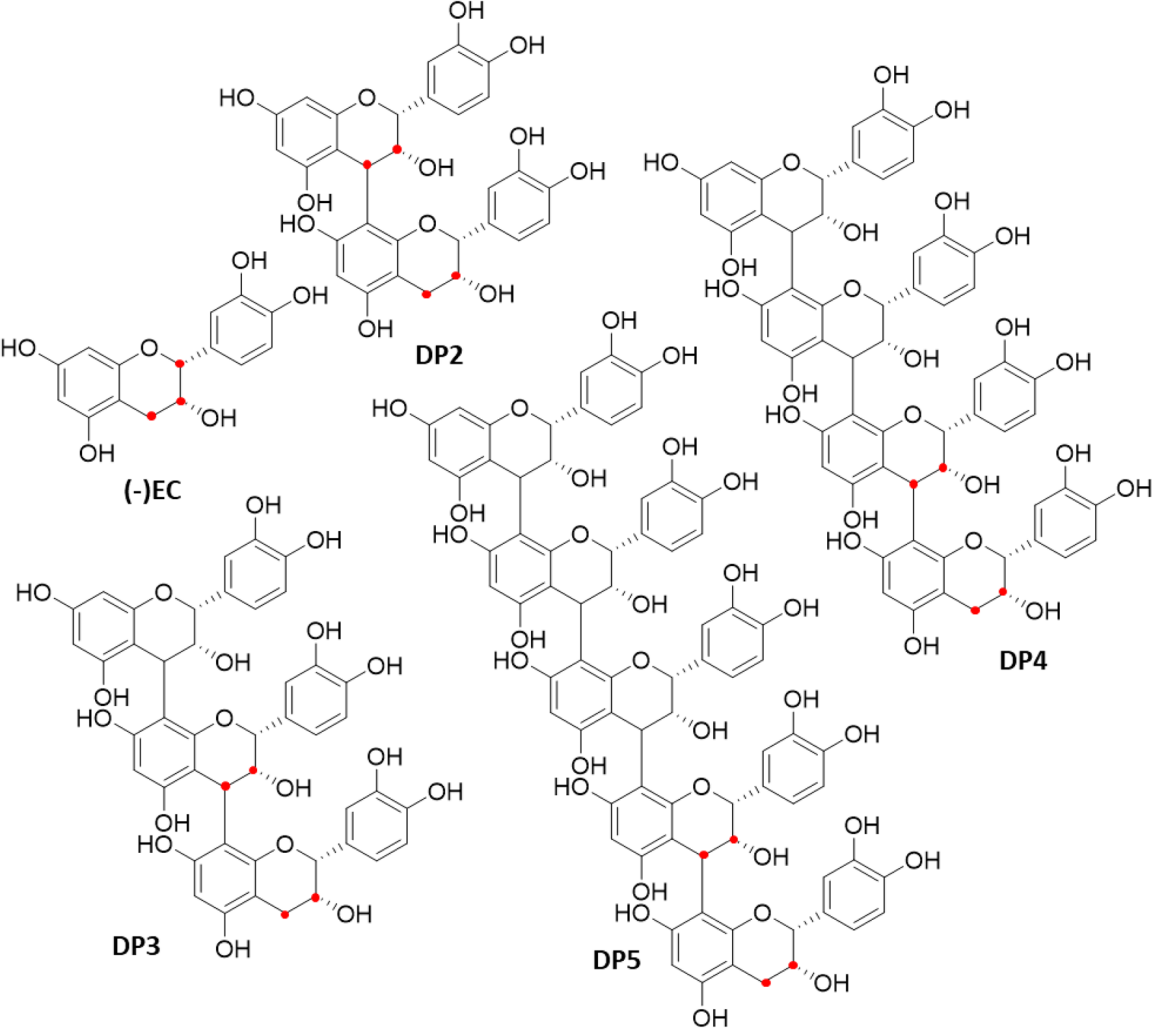
Structure of (−)-epicatechin and (−)-epicatechin oligomers (C4 → C8 B-type procyanidin) with degree of polymerization up to five (DP2–5), red dots represent carbon that are labelled with ^13^C on synthetic procyanidin standards. (structures drawn with ChemDraw Prime v17.1).

**Figure 2:**
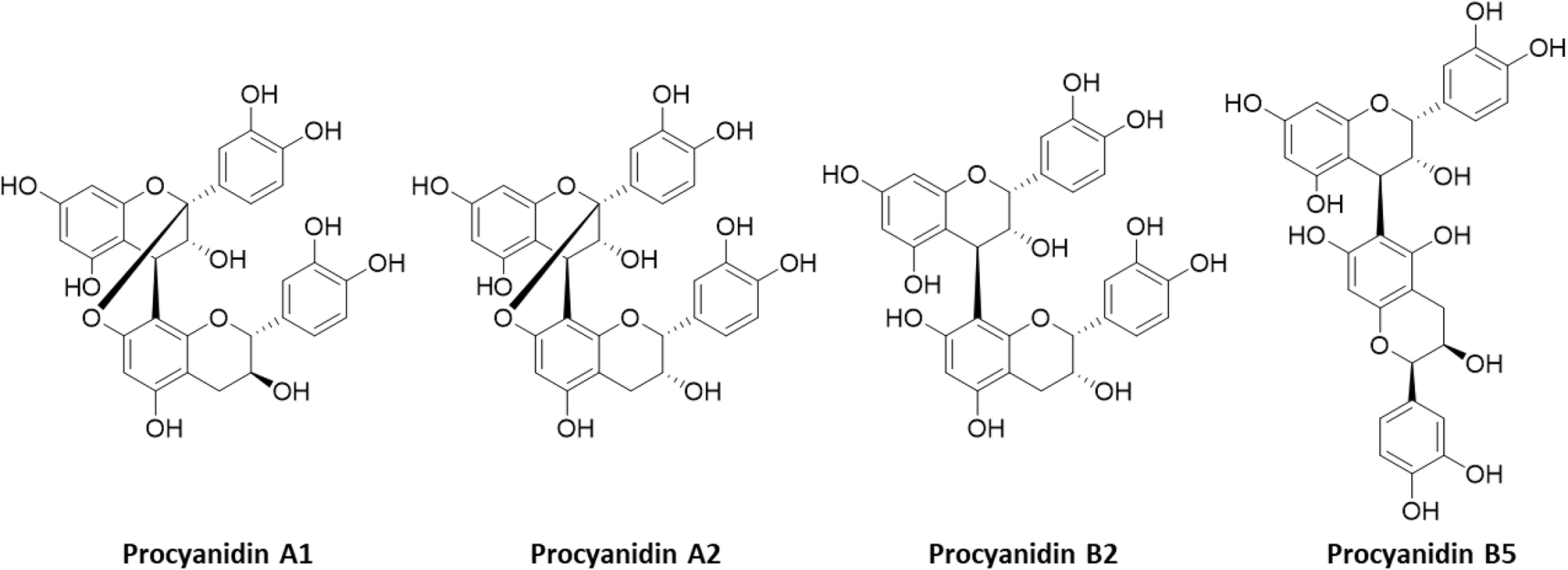
Structure of procyanidin A1, A2, B2 and B5 used for the evaluation of specificity. (structures drawn with ChemDraw Prime v17.1).

Analysis of flavanol monomers is relatively straight forward^10^; however, accurate quantitative analysis of procyanidins is a more difficult proposition^11–13^. While there are four naturally occurring flavanol monomers, namely (+)- and (−)-epicatechin and (+)- and (−)-catechin, these monomers can form oligomeric procyanidins with a variety of configurations (Fig. 1). Monomer sub-units can be linked via one or two covalent bonds, which, respectively, gives rise to B-type and A-type procyanidins (Fig. 2)^14^. In B-type procyanidins, found in products such as cocoa and apples^15^, the monomeric units predominantly have C4 → C8 linkages (e.g. procyanidin B2), although C4 → C6 bonds can also occur in lower abundance (e.g. procyanidin B5)^1,2^. In A-type procyanidins, found in peanuts and cranberries^15^, C4 → C8 links occur in conjunction with an O7 → C2 ether bond (e.g. procyanidin A1 and A2). This complexity results in a large variety of procyandin structures, which increase in parallel with the degree of polymerization (DP) as the number of sub-units increase.

HPLC–MS methodologies have been instrumental in identifying procyanidins of different sizes and structures, including oligomers containing other subunits such as gallocatechins and afzelechins^16^. Like optical detection-based methods, HPLC–MS is challenged by the lack of standards to quantify procyanidin oligomers. Approaches such as molecular relative response factors (MRRF)^15^, thiolytic degradation^17^ and external calibration^18^ have also been used to analyse flavanol and procyanidins in food. MRRF and thiolytic approaches alleviate the need for standards to support instrument calibration and achieve absolute quantitation. Direct calibration with standards is more reliable but costly, and in some cases impractical due to lack of commercial availability of the oligomers. Even though standards can be custom made, analysis by HPLC–MS often require the use of internal standards. Racine et al.^16^ proposed the use of a structural analogue which relies on the unverified assumption that all targeted procyanidins have the same behavior as the selected internal standard during extraction and MS ionization.

The use of HPLC–MS for the direct and accurate quantification of flavanols and procyanidins has not been reported and is dependent upon the the availability of analytical standards for method calibration and quantitative analysis. HPLC methods based on hydrophilic interaction chromatography (HILIC) separations have been used to analyse complex mixtures of procyanidins based on their DP. This simplifies quantifications as it reduces the number of analytical standards required to one per DP. As a proof of the validity of this approach, a reliable and transferable method based on fluorescence detection (FLD) was recently validated and accredited by AOAC (2020.05) to quantify procyanidins in cocoa-derived products^19^. Racine et al.^16^ reported the coupling of HILIC chromatography with MS^2^ detection and while this work is an important development for the quantitative analysis of procyanidins by DP, accuracy was not determined.

The current paper reports the development and validation of a versatile HPLC-MS^2^ method for the quantification of flavanols and procyanidins, using CFs as a model system. CFs were selected as their procyanidins have been characterized, with the predominant structures being C4–C8 B-type oligomers^11^. CF oligomeric fractions including DP2–7 isolated from the seeds of *Theobroma cacao* L. were used in combination with commercially-available procyanidin dimer standards, NIST cocoa extract reference material, and ^13^C-labeled procyandins (Fig. 2) to (1) develop HPLC-MS^2^ conditions for cocoa procyanidins, (2) study MS^2^ selectivity toward A-type and B-type procyanidins, (3) identify and compensate for matrix effects, and (4) validate and apply the method to commercial samples.

## Results and discussion

### Optimization of tandem mass spectrometry detection

Method development was initiated by studying collision induced dissociation (CID) of DP2–7 procyandidin standards isolated from cocoa (Table 1). The transformations identified on each target validate previously assigned fragments derived from complex mixtures analyzed by HPLC-MS^2^^15^. Retro Diels–Alder, quinone methide formation and heterocyclic ring fission were observed in addition to the neutral losses of carbon dioxide and water. The identification of selective MS^2^ transitions was the most challenging as B-type procyanidins present in cocoa show similar fragmentation patterns and interfering signals when forming multiple charged ions^18^. In this context, two transitions were selected for each target procyanidin (Table 1). In order to optimize method selectivity, neutral losses of carbon dioxide and water were not considered for the development of quantitative multiple reaction monitoring (MRM) traces. The first transition was used as a quantitative trace and the second was used as a confirmation ion. These transitions were selected to maximize sensitivity and selectivity (Table 1).

**Table 1:** Collison induced disssociation (CID) with optimal cone voltage (CV) and collision energy (CE) for cocoa flavanols and procyanidins with degree of polymerization (DP) of 1–7.

Compound	Parent ions (*m/z*) (CV)	Fragments (*m/z*) observed under CID	Ions selected for MRM (*m/z*) (CE)
DP1	289 (15)	245 227 221 205 203 179 161 151 125 109	109 (26) 205 (16)
^13^C_3_-DP1	292 (15)	248 125 109 223	109 (26)
DP2	577 (30)	559 451 425 407 381 289 287 245 125	425 ( 18) 451 (14)
^13^C_4_-DP2	582 (30)	429 411 366 292 248 181 163 125	429 (26)
DP3	865 (45)	739 713 695 577 451 425 407 289 287 245 125	287 (32) 577 (24)
^13^C_4_-DP3	869 (45)	529 455 410 291 125 244 343 428	291 (32)
DP4	1153 (45)	1108 956 865 821 577 425 413 407 381 289 287 245 125	287 (38) 575 (24)
^13^C_4_-DP4	1158 (45)	581 291 413 743 870 1005 987 951	581 (24)
DP5	720 (45)	907 863 720 695 575 450 407 289 245 125	407 (28) 695 (20)
DP6	865 (50)	1151 577 450 425 407 289 245 125	577.5 (20) 1151 (16)
DP7	1008 (40)	1440 1312 1296 1151 865 863 788 701 577 425 946 413 407 389 287 245 125	946 (18) 1268 (26)

After the identification of transitions for targeted CFs, ^13^C-labelled standards were included in the method. ^13^C-labelled structures are shown in Fig. 1. When analyzed individually, ^13^C-labeled DP1–4 procyanidins did not interfer with the detection of procyanidins with natural isotope abundance and vice-versa. This was not the case for ^13^C_4_-DP5 as interferences were observed and were associated with the formation of multiple charged ions which led to differences of *m/z* of only 2 (*m/z* 720 and 722, respectively, for natural isotope abundance and ^13^C_4_ labelled pentamers). In addition, the natural occurrence of heavy isotope is especially relevant with larger molecules, emphasizing the need to obtain ^13^C-labelled material with a DP higher than four with more than four labeled carbons. In the current study, only DP1–4 ^13^C-labelled reference compounds were available as internal standards for method development. Although financially constraining, ideal ^13^C-internal standards should be labelled on each oligomer monomeric unit. This approach would avoid the dilution of response between labelled and unlabelled fragments.

### Development of chromatographic conditions

**Figure 3:**
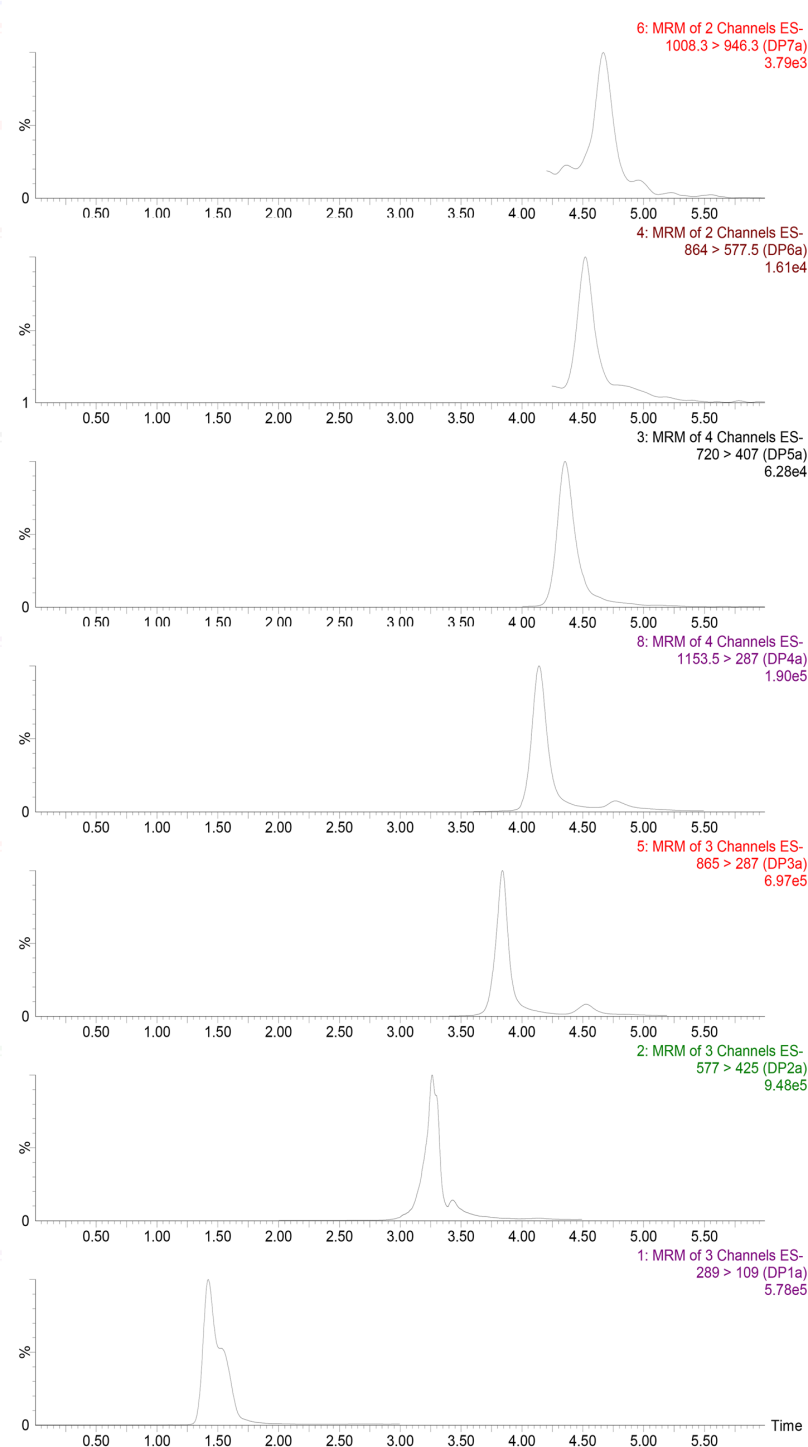
HPLC-MS^2^ extracted ion chromatogram of cocoa flavanols and procyanidins with degree of polymerization from 1 to 7 (bottom to top) in baking chocolate at endogenous concentrations. (Screenshot from Waters MassLynx 4.2).

HPLC was implemented to separate cocoa flavanol and procyanidin oligomers by DP using a column with a diol-modified sorbent. The impact of mobile phase composition, column temperature, column dimension, flow rate, injection volume and binary gradient parameters were evaluated. The official method of analysis relies on the separation of procyanidins using HILIC conditions. These conditions involve a high flow rate (1 mL/min) and a high concentration of acetic acid (2%) in the mobile phase and result in ion suppression that can be counteracted, in part, by using a post-column additive such as ammonium carbonate^18^. To limit the ion suppression phenomena, the current study focused on reducing the amount of acid entering the electrospray source. Use of a mobile phase containing 0.5% formic acid resulted in a slight decrease in response, but a tenfold improvement in the signal to noise ratio (S/N). In addition, MS^2^ detection allows for a slight overlap of signals leading to shorter run time than observed with FLD detection without compromising analytical performance. With these conditions, the flow rate was decreased to 0.3 mL/min and the gradient shortened to 10 min (when compared to 1 mL/min and 16 min for AOAC2020.05). A typical chromatogram is illustrated in Fig. 3.

### Comprehensive sample preparation

**Figure 4:**
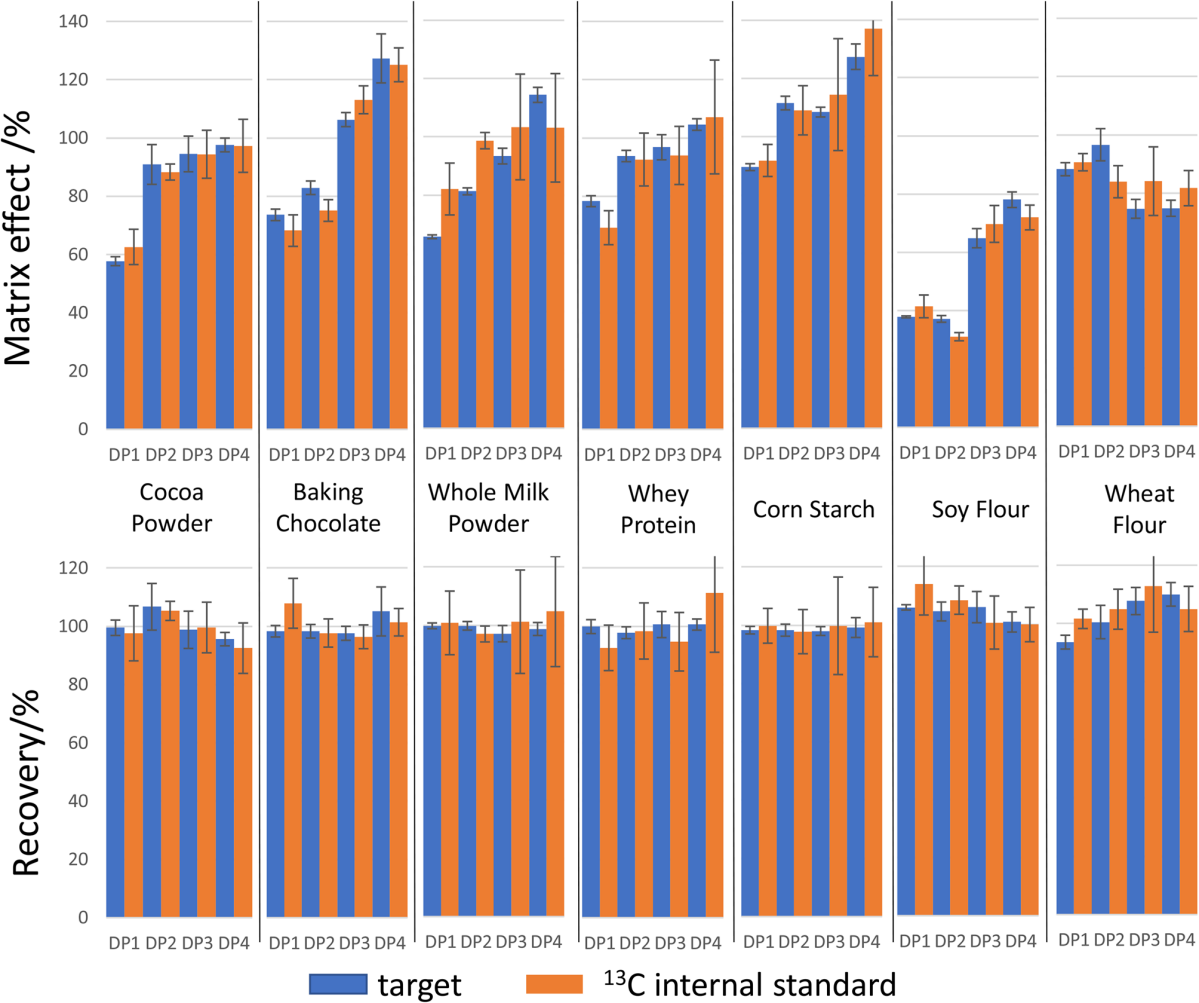
Matrix effect and recovery parameters for DP1–4 targets and respective ^13^C labelled internal standard in cocoa powder, baking chocolate, whole milk powder, whey protein, corn starch, soy flour and wheat flour. Blue and orange bars represent natural isotope abundance flavanol and procyanidins and ^13^C labelled (–)-epicatechin and epicatechin oligomers, respectively. Error bars show the standard deviation for five replicates. Recoveries (%) were determined by comparing signal area in sample spiked before and after extraction. Matrix effects (%) were determined by comparing signal area in sample solution spiked after extraction and standard solution.

Sample preparation was tailored for each of the seven model matrices, two of which were cocoa-based. These matrices were selected to provide a versatile sample preparation process that can accommodate different food/plant sample formats (with varying macronutrient composition) and, at the same time, provide insights in the quantification of flavanols and procyandins in sources other than cocoa. Detailed preparation for each of the seven matrices studied is oulined in Table S2. The evaluation of sample preparation began with the evaluation of recovery (comparison of content measured in sample spiked before and after extraction). Results are shown in Fig. 4 and highlight that there is no significant loss with the sample preparation conditions used. As reported in the literature, flavanol and procyanidins are efficiently extracted by solid phase extraction^15,20^. For cocoa, a mixture of acetone, water and acetic acid has consistently been shown to be efficient^18,21,22^.

Matrix effects were evaluated by comparing the responses of CF in NIST RM 8403, ^13^C-labelled (–)-epicatechin, B-type procyanidins in a matrix solution and a standard solution. For matrices containing endogenous levels of CF, target response was corrected for endogenous content. Matrix effect values determined were as anticipated. Signal suppression is often observed for peak eluting early chromatographic in analyses and are associated with co-eluting ion suppressing matrix components. Similarly, later eluting peaks (DP3+) can be slightly overestimated due the accumulation of charged apolar compound such as phospholipids. The identification of the origins of these matrix effects would require further experiment and was not further investigated in this work as the use of ^13^C labelled standards enabled their effects to accurately determined.

Matrix effects show two main trends (Fig. 4). First, responses were different across DPs which show that the use of a structural analogue as an internal standard cannot achieve accurate quantitative analysis of CF by HPLC-MS^2^. Second, ^13^C-labelled (–)-epicatechin and procyanidins showed comparable responses to their endogenous analogs isolated from cocoa. Thus, ^13^C-labelled flavanol and procyanidins, as shown in Fig. 1, can be used as internal standards alone or in combination with matrix match to compensate matrix effects.

Importantly, matrix effects were inconsistent across samples and across origin for a given matrices. For example, the matrix effect with three different brands of dark chocolate were evaluated at 47, 56 and 86%, indicating that the calibration curves built with one sample matrix blank would not be applicable to the other two. In this context, two approaches can be used to achieve accurate quantitative analysis. First, the matrix match approach does not involve the use of expensive ^13^C-labelled internal standards, but is less robust to interferences. The matrix match can be considered a reliable approach to achieve accurate measurement with the caveat that the matrix blank used to build calibration curve sample must be representative of the sample under study. This highlights the need to prepare multiple calibration curves when multiple types of samples are analyzed, even though these samples might be similar, for example, all be dark chocolates.

The second approach involves the use of stable isotope-labelled internal standards which is the most efficient and remains necessary when blank matrices are not available. To minimize the use of expensive ^13^C-labelled procyanidin internal standards, the matrix match approach was adopted when a blank matrix was available, while a ^13^C-labelled internal standards was used for cocoa samples for which a blank matrix was unavailable.

### Method validation

Method validation focused on determining accuracy, precision, sensitivity and selectivity parameters. Due to the availability of internal standards, experiments focused on CFs DP1–4. The quantification of CFs DP1–4 provides a significant input into material composition as it represent approximately 70% of total CFs (DP1–7 as measured using AOAC 2020,05) and covers the DP1–2 CFs for which dietary absorption in humans has been demonstrated^23^.

The first parameter evaluated was the method linearity and sensitivity. Method sensitivity is shown in Table S3 with the limit of quantification (LOQ) ranging from 10 to 50 ng/mL. These limits were determined as a S/N ≥ 10 were systematically below the lowest point of the calibration curve. Linearity was demonstrated by examining the coefficient of correlation of the calibration curve. A quadratic fit was used because a slight detector saturation was observed with DP1–2. The impact of matrix effect and interferences on the loss of sensitivity at higher concentration was ruled out as identical results were obtained in spiked sample matrix and standard solution. This is attributed to a higher concentration in the secondary standard used and a higher detector response than that observed for DP3–4. Regardless, coefficients of determination were systematically higher or equal to 0.99.

Precision and accuracy were assessed using a spike and recovery approach (Table 2). For each model matrix with an available blank, a stock solution was prepared and spiked with three different level of NIST RM 8403. The relative standard deviation across triplicate preparations determined precision, while the comparison of the measured value to the spiked amount estimated accuracy. For the two matrices containing endogenous procyanidins (cocoa powder and baking chocolate), four levels were prepared and only precision could be reported in the blank samples. As expected, precision improved as concentration increased. As shown by Table 2, intraday precision (%RSD) of up to 22.1% in LOQ and blank levels, while the highest %RSD was 5.7% at minimum quantifiable concentration (MQC) and highest quantifiable concentration (HQC) levels. Precision acceptance criteria were met for 78 of the 86 repeatability values measured. Precision for blank cocoa powder and blank baking chocolate was not considered as samples were prepared slightly below the lowest point of the calibration curve to allow accuracy verification at LQC (Lowest Quantifiable Concentration). Similarly, accuracy ranged from 90.9 to 125.4% at LQC level and 91.1 to 111.5% at MQC and HQC. Accuracy met acceptance criteria for 74 of the 84 values measured. Accuracy was consistently overestimated at LQC for the whole milk matrix which was also determined with the lowest level of precision. This suggest that sample preparation could be further optimized for dairy-based matrices, although the accuracy and precision achieved with the current conditions were acceptable. Interday precision showed similar trend with %RSD up to 20.0% at LQC level and 11.2% for MQC and HQC levels (interday precision data is available in supplementary information Table S4). These performances suggest that the HPLC-MS^2^ method develop can accurately and precisely measure flavanols and procyanidins.

**Table 2:** Percentage accuracy (%Acc) and intraday precision (%RSD) parameters for cocoa powder and baking chocolate using ^n^C labeled internal standards and for whole milk powder, whey protein, corn starch, soy flour and wheat flour using matrix match calibration.

	DP1		DP2		DP3		DP4	
	%Acc	%RSD	%Acc	%RSD	%Acc	%RSD	%Acc	%RSD
**Whole milk**								
LQC	115.1	9.5	115.6	7.7	114.0	4.7	125.4	9.8
MQC	108.7	2.3	109.5	1.8	106.7	4.4	108.7	4.7
HQC	103.3	4.2	102.2	1.0	101.0	2.7	107.6	3.2
**Wheat flour**								
LQC	92.5	9.4	94.0	3.8	99.4	2.6	92.4	4.2
MQC	109.5	3.8	103.2	1.6	105.6	1.7	102.7	1.8
HQC	111.5	3.1	105.7	2.1	107.2	0.8	105.0	3.6
**Corn starch**								
LQC	91.5	4.8	97.3	3.1	95.2	4.3	101.7	3.9
MQC	103.6	2.0	107.2	1.0	105.2	4.9	107.3	3.3
HQC	97.0	3.5	102.0	2.6	100.7	1.6	104.1	2.7
**Whey protein**								
LQC	90.9	8.8	97.2	2.2	104.1	3.7	105.1	4.6
MQC	99.4	4.8	97.7	1.2	100.6	4.7	104.4	2.9
HQC	99.4	2.7	100.0	1.1	101.1	4.1	104.1	2.6
**Soy flour**								
LQC	96.4	2.2	101.4	3.2	104.0	3.3	99.3	2.8
MQC	103.1	2.2	105.2	1.7	104.3	3.4	104.7	2.4
HQC	101.7	2.6	105.8	1.8	103.8	2.6	104.8	3.3
**Cocoa powder**								
Blank	na	5.1	na	22.1	na	21.9	na	19.1
LQC	101.9	8.1	103.9	10.9	99.3	4.9	100.9	5.6
MQC	100.4	5.7	101.4	3.5	96.6	1.3	100.8	2.7
HQC	100.3	3.6	100.7	1.4	92.5	2.0	102.1	2.1
**Baking chocolate**								
Blank	na	5.0	na	5.1	na	5.9	na	5.8
LQC	104.0	5.3	95.2	4.9	104.3	5.5	99.5	1.9
MQC	107.0	1.6	95.2	2.0	105.6	2.2	108.3	2.6
HQC	101.2	1.0	91.1	2.1	104.4	0.8	105.5	5.7

*LQC, MQC and HQC* low, middle and high quality check, *na* not analysed.

Method selectivity was investigated for two potential causes of interferences. The first source of interference investigated was between procyanidins with a different DP. With the formation of multiple charged ions, interferences are observed between targets. For example DP2 and DP4 show respective interferences with the double charged DP4 and double charged DP8. As a consequence, the separation of interfering oligomers by HPLC was essential to achieve the desired selectivity.

**Figure 5:**
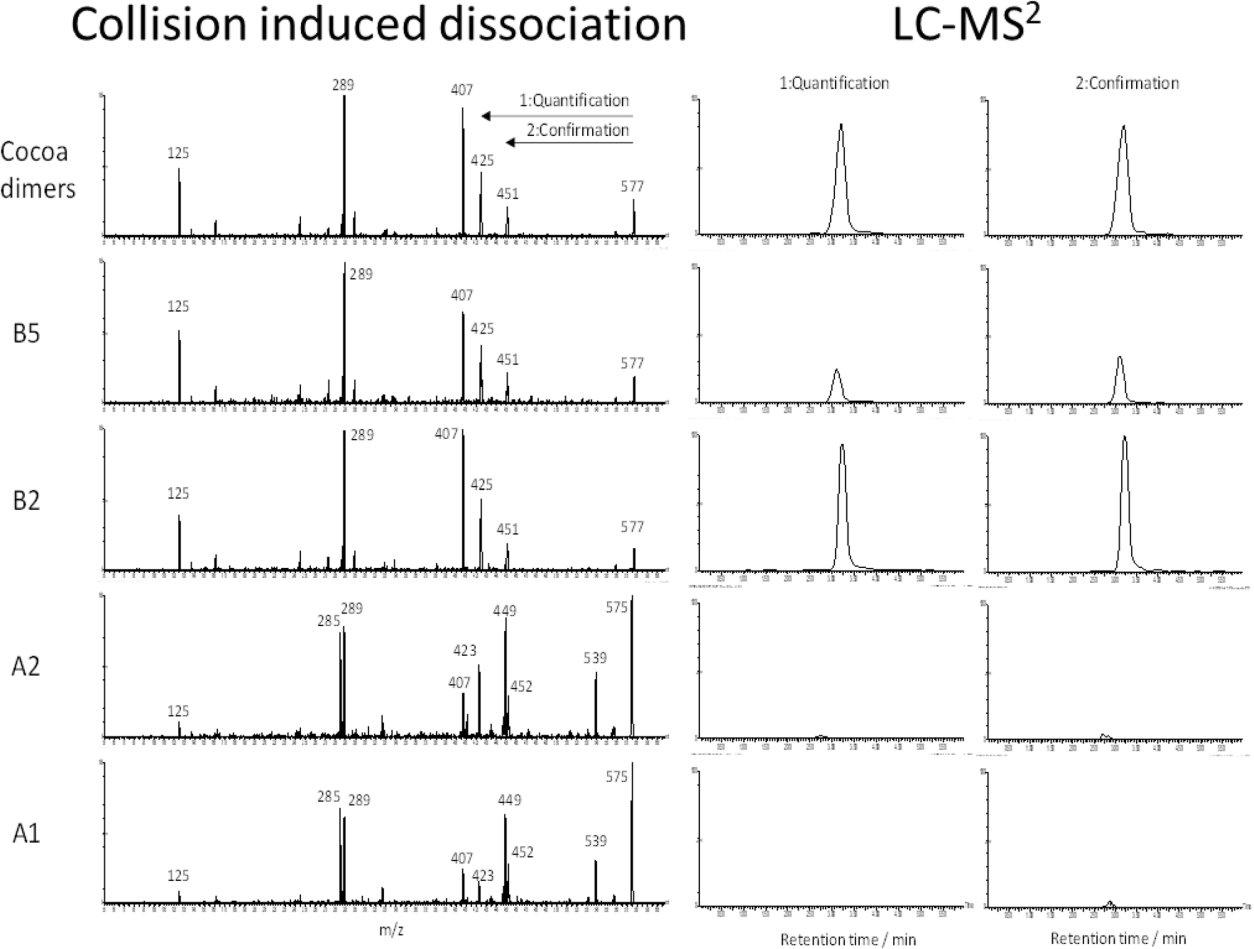
Left: MS^2^ collision induced dissociation with the multiple reaction monitoring (MRM) transitions used for quantification (*m/z* 577 > 407) and confirmation (*m/z* 577 > 425) of B-type procyanidin dimer. Right: HPLC-MS^2^ (MRM) of cocoa procyanidin with a DP2, procyanidin B5, procyanidin B2, procyanidin A2 and procyanidin A1 (top to bottom). Samples were all prepared at 10 µg/mL.

The second type of interference come from matrix components or other types of procyanidins that are not targeted by the method. A-type procyanidins are present in many botanicals, including cranberry and cinnamon. A- and B-type procyanidins have different CID patterns that can be leverage to develop the measurement of B-type procyanidins independently from A-type procyandins and vice-versa. To verify the selectivity of the method toward B-type procyanidins which are the only form identified in cocoa, procyanidins A1 and A2 (see structures in Fig. 5) were analysed and no signal was detected, confirming that cocoa is not a source of these specific procyanidins. Procyanidin B5 was also analyzed and the concentration was underestimated when compared to CF DP2 and isolated procyanidin B2 (Fig. 5). This indicated that the selectivity of this methodology was specific to C4 → C8 B-type procyanidins, but C4 → C6 B-type procyanidins could also contribute to the signal. This shows the limitation of quantative MS^2^ detection of procyanidins under the chromatographic conditions selected for this method. While reliable results can be achieved to differentiate A- and B-type linked species, MS^2^ detection does not allow discrimination between B-type procyanidins of the same DP. In order to achieve accurate results in the quantification of procyanidins in a given botanical, a reference material that matches the structural diversity of the targeted procyanidins in the sample represents a better alternative than using individual procyandins as analytical standards under the chromatographic conditions of this method (see Fig. 3).

Next, NIST RM 8403 was investigated as a secondary standard mixture. This material consists of a highly characterized cocoa extract that is already used as a secondary standard for the quantification of CF using a validated HPLC-FLD method^19^. Primary standards isolated from cocoa were used in a standard addition experiment to determine NIST RM 8403. Table S5 summarizes the concentration assigned by NIST on RM 8403 using HPLC-FLD^24^ and the estimates determined using HPLC-MS^2^ and standard addition of primary standards. Relative differences ranged from 2.1 to 4.2%, and < 3% for the sum of DP1–4. For three of the four oligomers, differences between the content determined and the value ascribed to NIST RM 8403 were below the standard deviation observed on the triplicate measurement. This suggests that NIST RM 8403 is an appropriate secondary standard calibrant for the determination of CF with a DP of up to four by HPLC-MS^2^. The NIST RM 8403 could be considered for the estimation of putative procyanidin concentrations in other botanicals with the caveat that the differences between procyanidin structures in the RM 8403 (cocoa) and the botanical of interest will lead to accuracy biases.

Stability of the samples was evaluated at 5 °C for autosampler and − 20 °C for freezer temperature. Internal ^13^C-labelled standards were not used for these measurements as they likely degrade at the same rate as the targets which would bias the evaluation of stability. Statistical analysis (ANOVA) was used to compare data acquired on each day. Results are presented in the Supporting Information (Fig. S2) for DP1–4 over 4 days. With the exception of DP3 in cocoa powder, samples were stable for 48 h, after preparation, for the two temperatures studied. DP3 in cocoa powder showed a statistically significant loss at both temperatures after 48 h but was stable for 24 h.

### Comparison to AOAC official method of analysis (AOAC2020.05)

**Figure 6:**
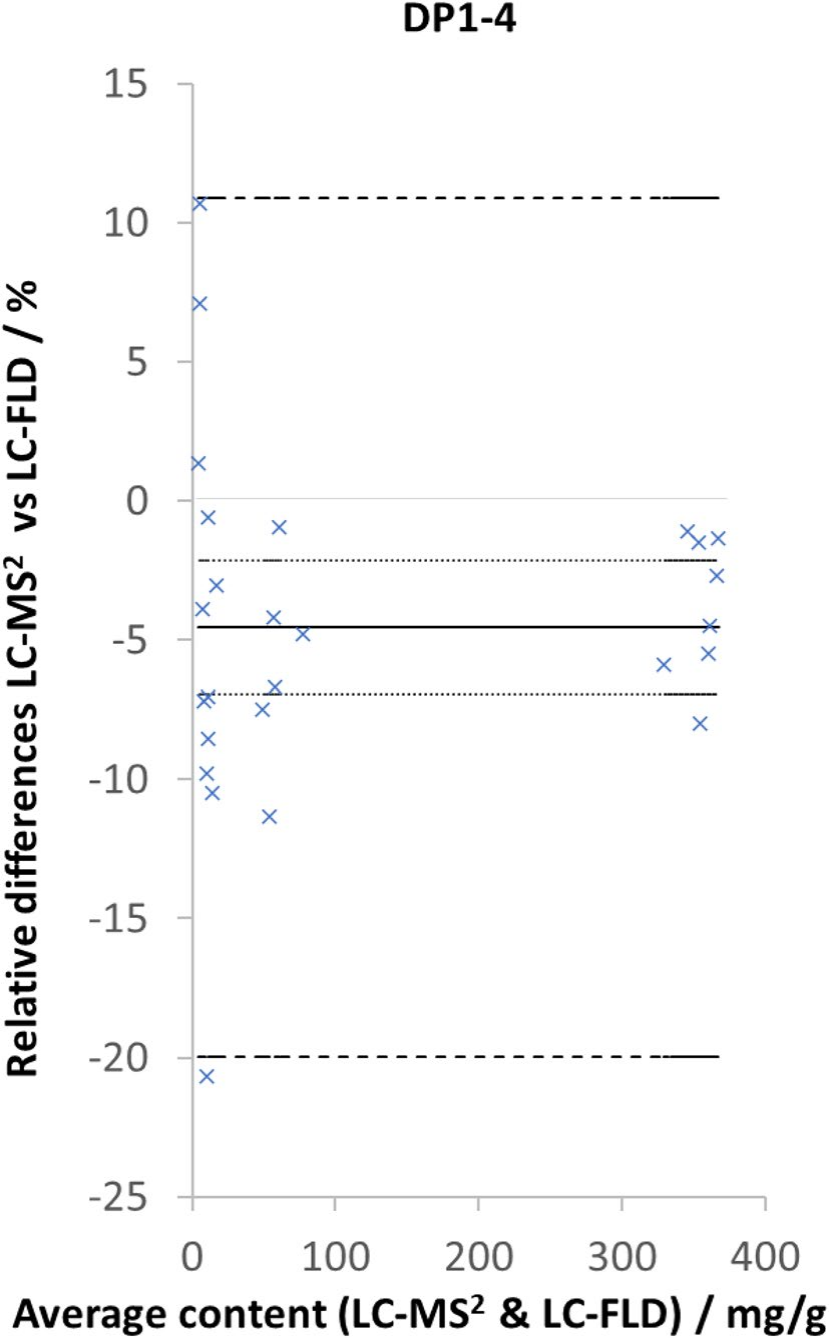
Bland–Altman plot of cocoa flavanol and procyanidin (DP1–4) relative difference between LC-MS^2^ in % testing and accredited HPLC-fluroescence detection (HPLC-FLD) testing as function of average DP1–4 content determined by both methods in mg/g. Solid black line represents the average relative difference between the two methods, the dotted line represent the 95% confidence on the average relative difference and the dashed black line represents the 95% confidence interval on a single estimate.

The method developed provides B-type specific quantitative analysis DP1–4 CFs. Because the HPLC-MS^2^ method was subjected to matrix effects that can impact on recovery, when possible, it is necessary to evaluate method accuracy through side-by-side comparison with an existing known reliable testing protocol, such as AOAC2020.05 for CF^25^. Although many other methods have been reported in the literature, only AOAC2020.05 has been fully validated and accredited as an Official Method of Analysis for the determination of CFs. Thus, the comparison of CF contents determined was limited to the HPLC-MS^2^ method discussed in this study and AOAC2020.05. To establish this comparison, a set of 26 samples, covering a wide range of concentration (3–540 mg/g) was analyzed using both methods. Figure 6 shows a Bland–Altman plot of the relative difference between DP1–4 determined with the HPLC-MS^2^ method and the AOAC accredited HPLC-FLD protocol. Similar plots are available in the Supplementary Information for the individual DPs (Fig. S3) and results are presented in Table S6. The relative difference average was 4.5%, indicating good agreement between the HPLC-MS^2^ and accredited testing methods. This slight underestimation of DP1–4 content can be attributed to the higher selectivity of MS^2^ detection. These results show that the results obtained by HPLC-MS^2^ are comparable to those determined through accredited testing and therefore demonstrate appropriate accuracy performance. Because of the scarcity of ^13^C-labelled procyanidins, the higher precision performances of fluorescence detection and the cost of instrumentation, HPLC-FLD would be the method of choice for routine analysis of CFs. However, when higher selectivity is sought and CF measurement is required to be specific to B-type procyanidins, HPLC-MS^2^ is the method of choice. Finally, HPLC-MS^2^ testing of CFs could be used to resolve disuputes between manufacturers and/or with regulatory bodies, or when the authenticity of a cocoa product needed to be verified by providing a more selective alternative to the existing methods relying on fluorescence detection.

## Conclusions

HPLC-MS^2^ represents a more selective quantification tool for cocoa flavanols and procyanidins than the HPLC-FLD method currently used for routine testing. Importantly, the MS-based method was shown to be in good agreement with the FLD-based method for the predominant procyanidin DP present in cocoa (DP 1–4), enabling the methods to be used in a complementary fashion. This method was capable of specifically quantifying B-type procyanidins even in the presence of A-type procyanidins of similar size, thus demonstrating a higher selectivity than fluorescent detection. The quantitation of DP1–4 provides an insight on approximately 60–70% of the cocoa flavanol and procyanidin content. More importantly, DP1–4 content includes flavanol monomers and procyanidin dimers which are the main compounds being absorbed and showing bioactivity. Future work should include the development of ^13^C-labelled procyanidin for higher DP (pentamer and larger) as these compounds may also have putative health benefits^26,27^.

The work presented in this manuscript focuses on cocoa and method performances were evaluated for CF determination. However, the information obtained in the development of the selective method for cocoa flavanol and procyanidins will be of value to studies with other botanicals. Flavanol and procyanidins are found in variety of foods and can be consumed in a healthy plant-rich diet. The development of such methods remains critical to further understand flavanol and procyandin distribution in the diet and to support investigations aiming at understanding the nutritional relevance of this goup of polyphenol bioactives from a variety of dietary sources.

## Material and methods

### Material and reagents

HPLC grade solvents, (−)-epicatechin [(−)EC)], procyanidins A1, A2, B2 and B5 standards were purchased from Sigma-Aldrich (Saint-Louis, MO, USA) and referred to as synthetic standard. (−)-[2,3,4-^13^C_3_]Epicatechin (^13^C_3_-(−)EC) was purchased from Cambridge Isotope Laboratories (Cambridge Isotope Laboratories, Inc Tewksbuy, MA, USA). Stable isotope labelled synthetic standard for procyanidins—procyanidin B2, (^13^C_4_-DP2); procyanidin C1, (^13^C_4_-DP3) and cinnamtannin A2, (^13^C_4_-DP4) were purchased from Analyticon Discovery with purity of ≥ 90% and no residual trace of unlabeled procyanidins. Carbon positions labelled with ^13^C are shown in Fig. 1. CF oligomeric fractions including DP_2–7_ isolated from the seeds of *Theobroma cacao* L. by Mars Wrigley Confectionary (Mars Inc, Hackettstown, NJ, USA), and purified and characterized (purities > 94%) by Analyticon Discovery (Analytical Discovery Gmbh, Potsdam, Germany) served as CF oligomer primary standards as previously reported^22^.

CF extract reference material (RM 8403)^24^, baking chocolate reference material (RM 2384), whole milk powder (RM 1549a), and soy flour (RM 3234) were acquired from the National Institute of Standard and Technology (NIST, US Dept. of Commerce, Gaitherburg, MD, USA). Corn starch, wheat flour, and whey protein were purchased at a local grocery store. Cocoa powder was supplied by Mars Symbioscience (Mars Inc, Germantown, MD, USA).

### Sample preparation

Seven matrices were selected to represent different foodstuff composition (Table S1). The distribution of the composition of these food matrices are presented in Fig. S1. Previously reported^19^ sample preparation was tailored for each of the seven foodstuff models using a combination of processing steps including defatting, solid/liquid extraction, protein precipitation and SPE clean-up which are summarized in Table S2 in the on-line Supplementary Information.

### Defatting of high fat matrices

Fat removal was performed for matrices with fat content above 10% by weight using solid–liquid extraction with hexane. Defatting was carried out by mixing 5 g of test material with 45 mL of hexane, sonicating at 50 °C, followed by centrifugation for 5 min at 1700 rcf. The hexane wash was decanted and the process repeated until the supernatant became clear. The hexane washings were combined and evaporated overnight at room temperature. The dry hexane residue was weighed to determine % fat content and enable fat correction of results. The deffated solid prepared for analysis.

### Solid/liquid extraction

CFs were extracted from solid samples with acetone:water:acetic acid (70/30/1, v/v) (AWAA). The mixture was vortexed, sonicated 5 min at 50 °C, and centrifuged for 5 min at 1700 rcf. The supernatant was either filtered prior to analysis or subjected to SPE purification.

### Protein precipitation

Water was added to samples which were vortexed, acetone:acetic acid (99.5/0.5, v/v) was added prior to sonication at 50 °C and incubation at − 20 °C for 20 min to allow complete protein precipitation. Samples were then centrifuged at 1700 rcf at room temperature for 5 min. The supernatant was either filtered prior to analysis or subjected to SPE purification.

### SPE purification

Solid phase extraction used a mixed-mode cation exchange cartridge (Oasis PRiME MCX 6 cc 150 mg) (Waters Coporation, Milford, MA, USA). A 1 mL volume of AWAA was used to condition the cartridge until ca. 2 mm remained on top of the sorbent. The cartridge was loaded with 2.5 mL of sample supernatent then as eluted until 2 mm remained on top of the sorbent. The sorbent was then washed with 12 mL AWAA which was collected and made up to a 25 mL volume with AWAA prior to analysis.

### Matrix effect and recovery

Matrix effect and recovery parameters were used to estimate sample preparation extraction and clean-up performances associated with HPLC-MS^2^ analysis. Matrix effect and recovery experiments were performed with a ratio of matrix to target ten times higher than the one described in the sample preparation section. This approach exacerbated matrix effect which facilitated their observation but can also lead to lower precision. Matrix effect and recovery were estimated using the a solution of standard, a matrix blank, a matrix spike both before and after extraction. Matrix effect was calculated as the ratio of the difference between the area response (HPLC-MS^2^) of matrix blank and matrix spiked after extraction to that of the standard solution. Recovery was estimated as the ratio of the response of the matrix spiked before and after extraction. Sample preparation was tailored to the compostion of each matrix (see Table S2).

### HPLC-MS^2^ optimization

The accurate analysis CFs by HPLC with MS^2^ detection is dependent upon the use of a chromatographic mobile phase without interferences from compounds with similar *m/z* ions to those produced by the compounds of interest. Full MS scans of the reference material (RM 8403) and primary standards were performed to identify *m/z* of parent compounds and optimal cone voltage conditions for CFs with different degrees of polymerization. The reference material and the primary standards were isolated from cocoa and, therefore, a C4 → C8 B-type linkage was expected to be the predominant structural feature of the targeted molecule. Cone voltage was evaluated from 10 to 80 V with an increment of 5 V and *m/z* scanned from 50 to 2000 Da. Daughter scans were then aquired for each target. Fragments were selected for their contribution to sensitive and selective detection. Collision energies were optimized from 10 to 80 V. To select the best compromise between sensitivity and selectivity, transitions that were unique to the targeted procyanidin MRM transitions were selected to achieve resolution against adjacent analytes in priority. MS^2^ parameters are summarized in Table 1.

### HPLC-MS^2^ analysis

A Water Acquity H-class liquid chromatograph linked to a tandem mass spectrometer (Waters Xevo TQS micro) with an electrospray source operating in negative mode was used for flavanol and procyanidin analysis. Chromatographic separations used a Waters Torus Diol Column (2.1 × 100 mm, 1.7 μm particle size, 130 Å pore size) fitted with an in-line filter. Column and autosampler temperatures were set to 50 °C and 5 °C, respectively. Samples, 2 µL, were injected and separated by a binary gradient with mobile phase A (acetonitrile:formic acid; 99.5:0.5, v/v) and mobile phase B (methanol:water:formic acid; 97:3:0.5, v/v). The solvent gradient at 0.3 mL/min was 0.0–0.4 min, 0%B, 3.0 min 45%B, 5.5 min, 95%B, 6.5 min 95%B, 6.6 min 0%B, and 10.0 min 0%B).

Negative mode electrospray ionization settings were as follows: desolvation gas flow was at 800 L/h, desolvation temperature was 500 °C, cone gas flow was 100 L/h, capillary voltage was at 3.2 kV, quadrupole low and high mass resolutions were lowered for the first and third quadrupole (LM1 resolution was set at 9.2, HM1 resolution was set at 12.0, LM2 resolution was set at 9.2, and HM2 resolution was set at 12.0) to accommodate the detection of multiple charged ions showing wider signal with low resolution detector. MRM detection conditions are described for DP1–7 in Table 1. However, due to accessibility to ^13^C labelled material, quantitative method development was only possible for DP1–4.

### Calibration

Cocoa extract reference material RM 8403 has been developed for the purpose of calibrating HPLC-FLD instruments used in AOAC Official Method of Analysis 2020.05. In this study, we used RM 8403 to calibrate a HPLC-MS^2^ instrument by preparing a serial dilution of NIST RM 8403. The stock solution was prepared by dissolving 40 mg of RM 8403 in a 50 mL flask with acetone:water:acetic acid (AWAA 70:30:1, v/v). This solution was diluted ten times to provide working standard #7. Working standard #7 was then diluted by pipetting 1.25, 2.5 and 5 mL in 10 mL volumetric flasks to obtain working standards 4–6 and 0.4, 0.8 and 1.5 mL in 25 mL volumetric flasks to obtain working standards 1–3. For cocoa samples, 1 mL of working standards 1–6 was transferred to autosampler vial and 10 µL of ^13^C internal standard solution (50 µg/mL) was added. Calibration curves were built for DP1–4 using the relative response of each target to its respective ^13^C internal standard, a 1/χ weighing function and a quadratic model. For samples with blank matrix available, a calibration curve was built in a similar manner, but with the use of ^13^C internal standard. Instead, a matrix match approach was preferred as a more cost-efficient option.

### Method validation

Method accuracy was determined through a spike and recovery approach at three levels, each prepared in triplicate. LQC (or low quality check) was the lowest level and was within 3 times the lowest level of the calibration curve. The intermediate level, MQC (or medium quality check), was placed in the middle of the calibration curve. The high quality check, HQC, had a concentration between the second to highest and highest level of the calibration curve. A solution of matrix was prepared at a concentration 4 times higher than what is recommended in Table S2, and 2.5 mL were transferred in 10 mL volumetric flasks. A solution of cocoa extract reference material was spiked at three levels and the volume adjusted to 10 mL. Accuracy was determined as the ratio between the measured concentration to spiked concentration. For cocoa powder and baking chocolate, the measured concentration was corrected for endogenous CF measured in unspiked samples.

Precision was assessed at three levels (four levels for matrices that contain endogenous CFs) and defined as the relative standard deviation (%RSD) across triplicate preparation within a single sequence (intraday) and three set of triplicate preparations analysed in three consecutive days (interday). Method validation acceptance criteria were defined by AOAC standard method performance requirements for flavanols in food and beverages. Acceptable repeatability was below or equal to 6% and acceptable accuracy between 85 and 108%.

Linearity was determined by the coefficient of determination (r^2^) higher or equal to 0.99. A quadratic fit was necessary due to the disparity in concentration and reponse across the degree of polymerization of the procyanidins. In these conditions, a slight saturation of the signal was observed at the highest concentration and led to using a quadratic model to fit the response = ƒ(concentration) curve.

Selectivity was assessed by analyzing other types of procyanidins. Cocoa procyanidins are predominantly B-type while other botanicals can contain A-type procyanidins or a mix of both A and B procyanidins. Commercially-available A-type procyanidins (procyanidin A1 and A2) were analyzed and showed no signal.

Stability was evaluated for the seven matrices over 4 days at autosampler (5 °C) and at freezer (− 20 °C) temperatures. Each sample was determined using a calibration curve solution prepared on the day of analysis. Percentage recoveries were calculated as the ratio of the content determined to the content determined on day 1, for DP1–4, for each matrix over a 3 day period. Statistical analysis (ANOVA) was performed to compare triplicate analysis performed on each day for each of the DP, matrix, temperature combination.

### Cross-validation

To estimate method reliability, performances were compared to those of the recently accredited testing (AOAC2020.05). Twenty six cocoa-based samples were analysed using the HPLC-MS^2^ method and the fluorescence-based protocol detailed in AOAC 2020.05. Sample matrices included a wide-range of commercially-available cocoa-based ingredients including cocoa powder, dark chocolate, baking chocolate, dietary supplement drink mixes, dietary supplement capsules and dietary supplement cocoa extract ingredients. These samples had CF contents ranging from ca. 3 to 500 mg/g (DP1–7 per AOAC2020.05). Given the limited number of radiolabeled standards, DP1–4 contents measured and compared across the the two methods. Method differences were assessed by modeling CF content determined by HPLC-MS^2^ as a function of CF content determined with HPLC-FLD.

## Supplementary Information

Supplementary Information 1.
