# Supplementary material for: Development and validation of HPLC-MS2 methodology for the accurate determination of C4–C8 B-type flavanols and procyanidins
Source: Sci Rep. 2021 Jul 20;11:14761. doi: 10.1038/s41598-021-93993-0 (PMC8292358; doi:10.1038/s41598-021-93993-0)
Supplement: Supplementary file 1 — Supplementary Information 1. [file 41598_2021_93993_MOESM1_ESM.docx]

Supplementary Information

Development and validation of HPLC-MS^2^ methodology for the accurate determination of C4-C8 B-type flavanols and procyanidins

Table S1. Formulation of sample composite prepared to support method validation experiments. Ratios (weight to weight) indicate the ratio between NIST cocoa flavanol extract RM 8403 and each model food matrix

| Matrix | ratio |
| --- | --- |
| Corn starch | 1:8 |
| Whey protein | 1:30 |
| Whole milk powder | 1:32 |
| Wheat flour | 1:120 |
| Soy flour | 1:100 |

Table S2. Matrix sample preparations.

| **Matrix** | **Weight (mg)** | **Volume (mL)** | **Defatting** | **Protein precipitation** | **Sonication** | **Centrifugation** | **SPE** | **Dilution** | **Filtering** |
| --- | --- | --- | --- | --- | --- | --- | --- | --- | --- |
| Cocoa powder | 100 | 25 | Hexane | none | 5 min; 50 ℃ | 5 min; 1700 rcf | MCX PRiME | 25 mL | none |
| Baking Chocolate | 100 | 25 | Hexane | none | 5 min; 50 ℃ | 5 min; 1700 rcf | MCX PRiME | 25 mL | none |
| Whole milk powder | 100 | 25 | Hexane | Acetone | 5 min; 50 ℃ | 5 min; 1700 rcf | MCX PRiME | 25 mL | none |
| Whey protein | 20 | 25 | none | Acetone | 5 min; 50 ℃ | 5 min; 1700 rcf | none | none | PTFE 0.45 µm |
| Corn starch | 20 | 25 | none | none | 5 min; 50 ℃ | 5 min; 1700 rcf | none | none | PTFE 0.45 µm |
| Soy flour | 20 | 25 | none | Acetone | 5min; 50 ℃ | 5 min; 1700 rcf | none | none | PTFE 0.45 µm |
| Wheat flour | 20 | 25 | none | none | none | none | none | none | PTFE 0.45 µm |

Table S3. Limit of quantification (LOQ), lower calibration point (LCP) and linearity (r^2^) for a DP1-4 in cocoa extract.

|  | DP1 | DP2 | DP3 | DP4 |
| --- | --- | --- | --- | --- |
| LOQ (ng/mL) | 25 | 10 | 25 | 50 |
| LCP (ng/mL) | 165 | 115 | 125 | 105 |
| r^2^ | ≥0.99 | ≥0.99 | ≥0.99 | ≥0.99 |

Table S4. Interday precision (%RSD) parameters for cocoa powder and baking chocolate using ^13^C labeled internal standards and for whole milk powder, whey protein, corn starch, soy flour and wheat flour using matrix match calibration. Low, Middle and High Quality Check (LQC, MQC and HQC). Three replicates per level and per day were acquired for three consecutive days.

|  | | DP1 | DP2 | DP3 | DP4 | |  |
| --- | --- | --- | --- | --- | --- | --- | --- |
|  |  | %RSD | %RSD | %RSD | | %RSD | |
| Whole Milk | LQC | 9.1 | 16.9 | 14.0 | | 10.4 | |
|  | MQC | 4.9 | 9.2 | 9.4 | | 10.0 | |
|  | HQC | 6.9 | 6.3 | 6.2 | | 5.4 | |
| Wheat Flour | LQC | 7.7 | 10.8 | 17.4 | | 18.4 | |
|  | MQC | 7.5 | 7.5 | 8.7 | | 7.7 | |
|  | HQC | 4.1 | 4.4 | 4.5 | | 5.9 | |
| Corn Starch | LQC | 10.8 | 13.2 | 8.5 | | 16.0 | |
|  | MQC | 8.0 | 7.7 | 11.1 | | 6.5 | |
|  | HQC | 3.5 | 6.7 | 6.9 | | 4.7 | |
| Whey Protein | LQC | 11.9 | 15.1 | 11.1 | | 20.0 | |
|  | MQC | 5.5 | 11.5 | 5.7 | | 8.6 | |
|  | HQC | 5.0 | 4.3 | 7.3 | | 6.1 | |
| Soy Flour | LQC | 8.5 | 10.4 | 10.2 | | 17.1 | |
|  | MQC | 7.9 | 6.6 | 8.4 | | 11.2 | |
|  | HQC | 4.6 | 5.7 | 6.3 | | 5.7 | |
| Cocoa powder | Blank | 7.1 | 8.1 | 7.5 | | 8.7 | |
|  | LQC | 7.8 | 6.7 | 8.7 | | 7.7 | |
|  | MQC | 4.9 | 4.1 | 6.7 | | 5.6 | |
|  | HQC | 4.3 | 4.4 | 4.3 | | 5.1 | |
| Baking chocolate | Blank | 16.4 | 16.6 | 16.9 | | 18.7 | |
|  | LQC | 7.9 | 6.1 | 10.3 | | 8.8 | |
|  | MQC | 5.6 | 3.6 | 6.1 | | 8.6 | |
|  | HQC | 1.9 | 2.6 | 5.5 | | 4.2 | |

Table S5. Determination of NIST RM 8403 by standard addition of flavanol and procyanidin primary standards followed by HPLC-MS^2^ analysis.

| Flavanols | NIST RM 8403 (mg/g) | | LC-MS^2^ (mg/g ± SD) | Difference | |
| --- | --- | --- | --- | --- | --- |
|  | Content | Uncertainty |  | mg/g | % |
| DP1 | 114.9 | 1.0 | 111.8 ± 4.4 | 3.1 | 2.7 |
| DP2 | 82.6 | 1.4 | 79.9 ± 2.8 | 2.7 | 3.3 |
| DP3 | 87.4 | 1.9 | 85.6 ± 4.6 | 1.8 | 2.1 |
| DP4 | 74.0 | 1.5 | 70.9 ± 0.4 | 3.1 | 4.2 |
| DP5 | 62.7 | 1.3 | 57.8 ± 1.4 | 4.9 | 7.8 |
| DP6 | 48.4 | 1.1 | 43.8 ± 1.4 | 4.6 | 9.5 |
| DP7 | 36.7 | 1.0 | 39.6 ± 2.3 | −2.9 | −7.9 |
| Total | 506.7 | na | 489.3 | 17.4 | 3.4 |

Table S6. Average relative differences for cocoa flavanols contents determined with this LC-MS^2^ method and AOAC2020.05 for individual DP and the sum of DP1-4 with 95% confidence on the average relative difference and on a single point estimate.

|  | DP1 | DP2 | DP3 | DP4 | Total DP1-4 |
| --- | --- | --- | --- | --- | --- |
| Average difference (%) | −3.9 | −2.4 | −8.3 | −6.6 | −4.5 |
| Standard deviation dev difference (%) | 9.0 | 15.9 | 6.4 | 9.0 | 6.0 |
| Degree of freedom | 25.0 | 25.0 | 25.0 | 25.0 | 25.0 |
| t value | 2.06 | 2.06 | 2.06 | 2.06 | 2.06 |
| Standard error (%) | 1.8 | 3.1 | 1.2 | 1.8 | 1.2 |
| Confidence (%) | 3.6 | 6.4 | 2.6 | 3.7 | 2.4 |
| Lower confidence interval on mean (%) | −7.6 | −8.8 | −10.9 | −10.2 | −7.0 |
| Upper confidence interval on mean (%) | −0.3 | 4.1 | −5.7 | −2.9 | −2.1 |
| Lower confidence interval on measure (%) | −27.1 | −43.4 | −24.7 | −29.9 | −20.0 |
| Upper confidence interval on measure (%) | 19.3 | 38.6 | 8.1 | 16.8 | 10.9 |


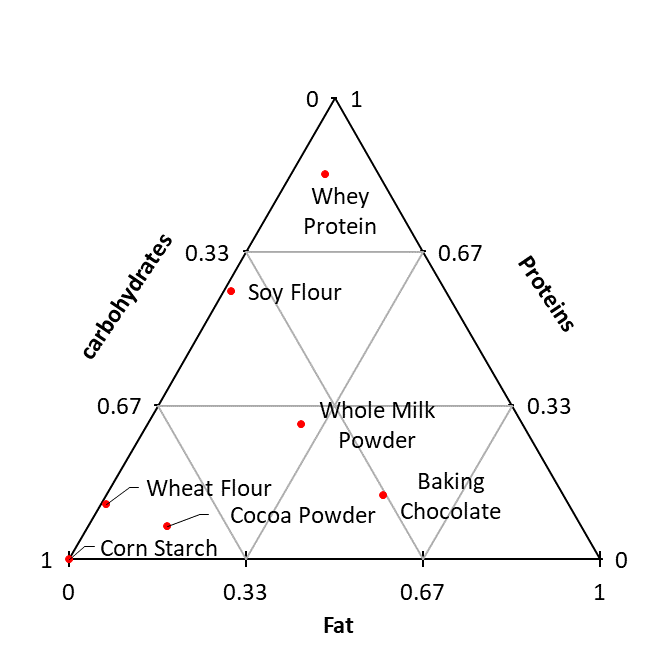


Figure S1. Food triangle scheme showing matrices distribution based of food composition


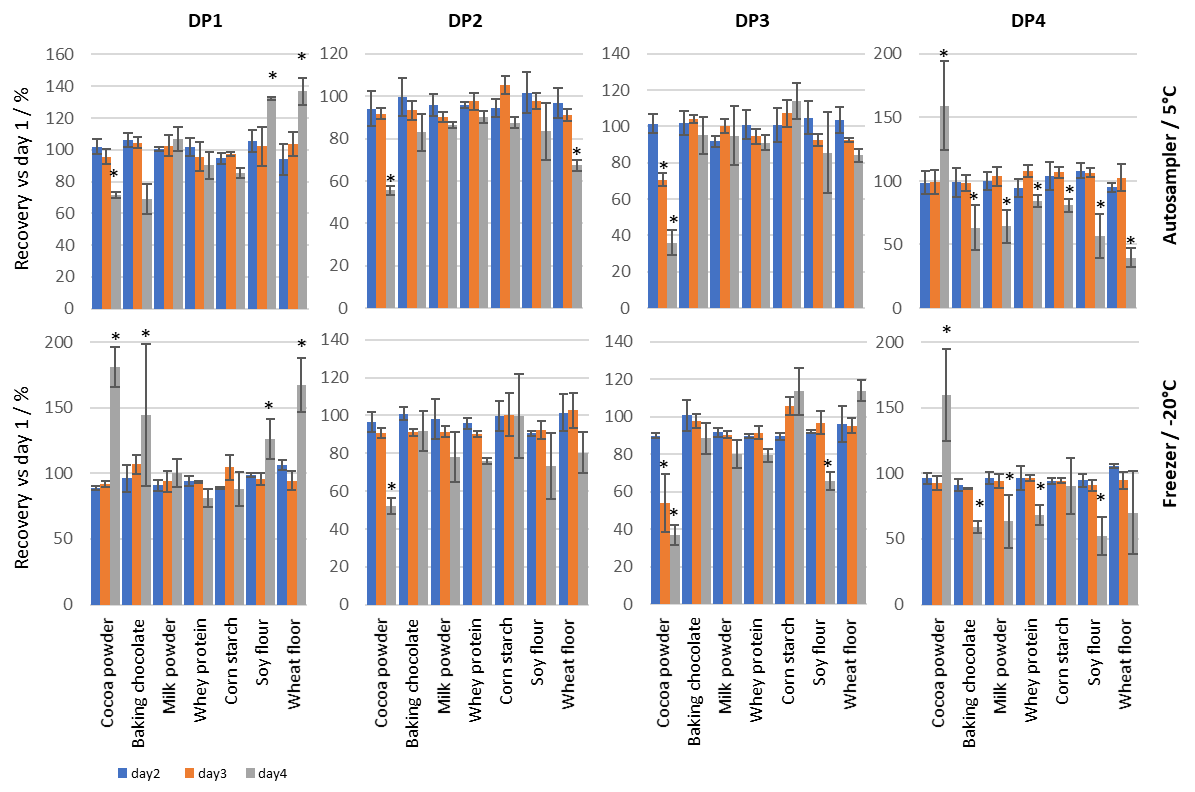


Figure S2: Stability of DP1-4 in 7 model matrices after incubations at 5 °C and 20 °C for 24h, 48 h and 72 h. Stability was assessed as the recovery (%) expressed relatively to content determined in samples before incubation. Data are expressed as mean values ±SD (n=3). * p<0.05 compared to recovery before incubation (ANOVA).


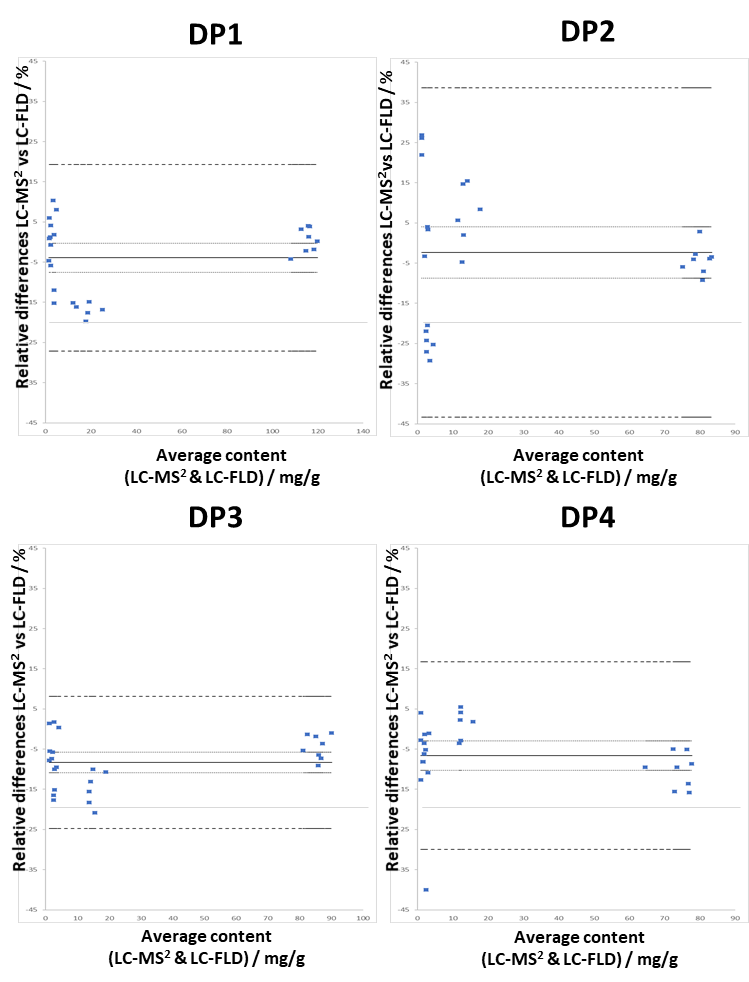


Figure S3: Bland-Altman plots for relative differences for cocoa flavanols contents determined with this HPLC-MS^2^ method and AOAC2020.05 as a function of the average of the content determine with two methods for individual DP and the sum of DP1-4. Solid black line represents the average relative difference between the two methods, the dotted line represent the 95% confidence on the average relative difference and the dashed black line represents the 95% confidence interval on a single estimate.
